# The Effect of Insight Questions Inventory and Visual Support Strategies on Carer-Reported Quality of Life for Children With Cerebral Palsy and Perceptual Visual Dysfunction in Nigeria: A Randomized Controlled Trial

**DOI:** 10.3389/fnhum.2021.706550

**Published:** 2021-11-16

**Authors:** Roseline E. Duke, Torty Chimaeze, Min J. Kim, Soter Ameh, Kathryn Burton, Richard Bowman

**Affiliations:** ^1^London School of Hygiene and Tropical Medicine, International Center for Eye Health, University of London, London, United Kingdom; ^2^Children’s Eye Center, Department of Ophthalmology, University of Calabar Teaching Hospital, Calabar, Nigeria; ^3^Department of Pediatrics, Pediatric Neurology, University of Calabar Teaching Hospital, Calabar, Nigeria; ^4^Department of Community Medicine, University of Calabar Teaching Hospital, Calabar, Nigeria; ^5^Cambridgeshire Community Services, Oxfordshire, United Kingdom

**Keywords:** cerebral palsy, cerebral visual impairment, perceptual visual disorders, insight questions inventory, visual support strategies

## Abstract

Structured clinical history question inventories have previously been used to try and elicit symptoms of perceptual visual dysfunction (PVD) in children with cerebral palsy (CP) in different settings. Earlier studies have suggested that PVD may affect quality of life and specific habilitational strategies, linked to inventory responses, may improve quality of life. Through an RCT, based on a community based sample of children with CP in Cross River State, Nigeria, we aimed to determine if a structured history inventory such as the Insight question inventory (IQI) and associated tailored visual support strategies (IQI VSS) for the management of those children who have PVD, can improve quality of life and is superior to standard therapy. Children with CP were recruited by the key informant method and confirmed by clinical examination. The parent reported IQI was used to identify children with PVD. Primary outcome measures were both Pediatric Quality of Life 4.0 Generic (PedsQL 4.0 Generic) and Pediatric Quality of Life 3.0 Cerebral Palsy (PedsQL 3.0 CP) scale scores. Children were enrolled with a parallel arm allocation to either IQI and IQI VSS or to standard therapy for CP. Children were followed up for 6 weeks with weekly phone call session and the questionnaires repeated at the end of the 6 weeks’ period. Results show that the children in the treatment group (*n* = 191) showed no significantly different change between baseline and follow up in quality of life (PedsQL 4.0 Generic *p* = 0.943: and PedsQL-CP 3.0 *p* = 0.287), compared to the control group. There was suggestion of a better improvement (*p* = 0.035) in the PedsQL 3.0 CP subscale of speech and communication for the intervention group. The use of IQI VSS for the treatment of PVD in children with CP in this population does not show any superiority over current standard CP management in terms of overall quality of life. However, there was some evidence of improvement in quality of life in the area of speech and communication. Further research and refinement of these management method is required.

**Clinical Trial Registration:** www.ClinicalTrials.gov, identifier [PACTR20161200188] 6396.

## Introduction

Cerebral palsy (CP), is the most common neurologic and motor disability in children globally (Surman et al., 2006). CP describes a group of permanent disorders of the development of movement and posture causing activity limitation, which are attributed to non-progressive disturbances that occurred in the developing fetal or infant brain (Rosenbaum et al., 2007). The motor deficits are often accompanied by disturbances of sensation, perception, cognition, communication, behavior, epilepsy, secondary musculoskeletal problems and nutrition (Rosenbaum et al., 2007).

World Health Organization, through the International Classification of Functioning, Disability and Health (ICF), clarified the understanding of CP in relation to intervention options and differentiated functioning problems, participation problems and disability. It is suggested that interventions should aim at maximizing a child’s independence in daily activities and community participation, while also focusing on optimizing children’s environment. In addition, a goal-based approach and (Zihl and Dutton, 2015), patient centeredness based on choice of interventions guided by what would best help the family achieve their goals, is recommended (Novak et al., 2013).

Visual impairment in children with CP varies in nature and severity and its prevalence ranges from 40-50% of children in different studies (Ego et al., 2015). Visual impairment in CP is often directly associated with the same brain injury which causes the motor problems. This is generally termed cerebral visual impairment (CVI) (Sakki et al., 2018), commonly affecting children with CP but also children with other neurodevelopmental diagnoses such as epilepsy and hydrocephalus. CVI can cause problems with ‘basic’ vision such as visual acuity or visual field or affect ‘higher’ visual perception or cognitive vision such as ability to see moving targets, to pick out a target of interest from a complex scene, visual control of body movement or object recognition (Dutton, 2011). This latter group of behavioral symptoms of abnormal higher-order visual processing can be termed perceptual visual dysfunction (PVD), part of the CVI spectrum.

It was previously shown in India, Bangladesh and more recently in the United Kingdom (Mitry et al., 2016; Philip et al., 2016; Tsirka et al., 2020), that this latter group of symptoms of higher visual processing problems or PVD are commonly present in children with CP; we have previously published a detailed visual assessment of the children recruited for this trial showing high rates of visual pathology (including 46% PVD measured objectively and 49% CVI, dropping to 16% if optic atrophy excluded) (Duke et al., 2020).

Previous work has suggested that PVD can be effectively assessed by a structured clinical history question inventories, including the insight question inventory (IQI) (Macintyre-Beon et al., 2012; Mitry et al., 2016; Philip, 2017; Tsirka et al., 2020). IQI scores have been shown to have internal reliability, and to discriminate between children diagnosed with CVI and healthy aged matched volunteers (Macintyre-Beon et al., 2012; Philip and Dutton, 2014); they have also been shown to correlate with neuropsychological tests of visual perception (Tsirka et al., 2020), and predict quality of life in children with CP independent of other predictors such as visual acuity and degree of motor impairment (Mitry et al., 2016).

The IQI provides in-depth information about the aspects of daily living activities that children struggle with. The current 52 item inventory, Insight Questions Inventory tests 6 domains of vision namely, visual field, perception of movement, visual guidance of movement, visual search, visual attention, recognition and navigation, which, in addition to visual field, test visual perception, either dorsal (occipito-parietal) stream processing (visuo-motor control, processing moving targets and processing large amounts of visual information at once) or ventral (occipito-temporal) stream processing (person and object recognition).

Previous work indicated that considering dorsal and ventral stream as 2 factors explained 63% of variance of IQI scores between patients (Philip et al., 2016). In addition to diagnostic information, a simple software program links each question inventory response to a specific group of tailored visual support strategies (VSS) appropriate to that question, so that after completing the inventory each child/family has a set of tailored visual support strategies (IQI VSS) for that particular child. An example of the IQI and Visual support strategy can be seen in question 11, which asks “Does your child bump into door frames or partly open doors (left/right/both)”? Corresponding tailored visual support strategies would include suggesting that the caregivers would give extra hints. For example, “There is a door coming up in a few steps.” Another recommendation would be to replace doors with a beaded curtain.

A recent hospital based longitudinal study investigated the impact of the IQI VSS linked to the inventory responses on functional vision and quality of life (Tsirka et al., 2020). Children were followed up 6 months after receiving the IQI VSS and improvements were seen in both qualities of life and functional vision compared with baseline pre-intervention assessments but there was no control group.

We aimed to test whether this approach would be effective for a community based sample of children with CP in Cross River State, Nigeria by means of a randomized controlled trial. We did not screen for CVI or PVD within the CP sample but recruited all children with CP. The rationale for this was that we predicted high rates of PVD detectable with IQI and that, even for those without formal PVD, almost all children with CP would have at least one positive response to IQI and therefore receive at least one IQI VSS. The aim was to test the impact of IQI VSS on children with CP rather than those with CP *and* CVI.

Our previous work has suggested that PVD (measured by IQI) adversely affects quality of life, (Mitry et al., 2016) and that IQI VSS improve quality of life (Tsirka et al., 2020), using the PedsQL 4.0 Generic module which assesses domains of physical activities, social, emotional and school functions. The PedsQL 3.0 CP module, is designed to be used by children with CP to detect changes arising from this condition or factors associated with it, with subdomains which include daily activities, movement and balance, fatigue, pain, school functions, eating and speech (Varni and Said, 2006; Varni et al., 2006; Viehweger et al., 2008). Peds QL 4.0 Generic and PedsQL 3.0 CP assessments can be based on parent report, child reports or proxy reports (Varni et al., 2007; White-Koning et al., 2007). Since PedsQL3.0 CP is specifically designed for children with CP but has not been previously tested in relation to CVI or PVD we decided to use both PedsQL 4.0 Generic and PedsQL 3.0 CP modules as primary outcomes for this trial.

## Materials and Methods

**FIGURE 1:**
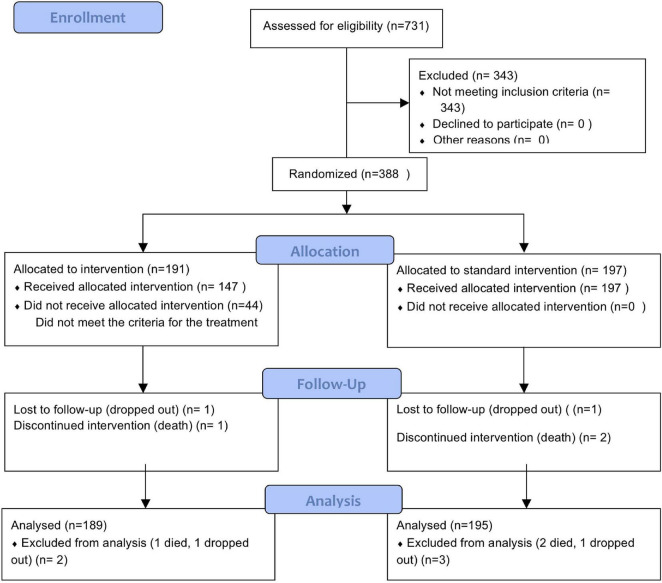
Shows the consort flow chart.

This was a parallel group, double blind clinical trial, with a superiority design (Figure 1). Recruitment took place between December 2016 to December 2018. Details of the trial methodology have been published and are briefly summarized here (Duke et al., 2019).

### Participants

This prospective population study was conducted in 18 local government areas in Cross River State, Nigeria. Recruitment took place over 12 months, using the key informant method (Murthy et al., 2014). In the first stage, a population based sample of children suspected to have CP were identified by the key informants with the use of the Ten questions Questionnaire and CP picture chart (Muhit et al., 2007). In the second stage, children had a comprehensive history taken and detailed examination, including neurological examination and confirmation of the diagnosis and classification of CP by a pediatric neurologist who used the diagnostic criteria for CP (Surveillance of Cerebral Palsy in Europe, 2000). Detailed visual assessment was performed and the results have been published including the Insight question inventory for PVD (Duke et al., 2020).

Eligible children were those confirmed to have CP who consented to be recruited into the trial (Rosenbaum et al., 2007). Data on all eligible children address and phone number(s) of carers was entered into a password protected database. Each child was allocated a unique identification number.

### Assessment of CVI/PVD

For the purpose of the trial, symptoms of PVD were behaviorally ascertained by the use of the Nigerian Version IQI, a 52-item symptoms based inventory, (Supplementary Material 1), derived through linguistic translation of the British version of the insight questions inventory, and which was administered to each carer. There are 6 sections. Responses to each question was in accord with a 5-point Likert scale (1-5) to describe whether a child has problems: never, rarely, sometimes, often or always including “not applicable” respectively. The questions in each section are designed to identify CVI/PVD through asking about visual tasks involving both the dorsal (sections 1-5) and ventral (section 6) visual streams. Any subject who answered “sometimes”, “often” or “always” to at least one question (out of 52) would be considered to have PVD and would receive at least one strategy. Questions with more than half of respondents reporting “n/a” were excluded (Mitry et al., 2016).

### Assessment of Speech and Communication

The Communication Function Classification Scale (CFCS) assessed the full activity of communication in five levels between a familiar person and the child (Virella et al., 2016). We referred to children as having communication impairment if CFCS was level 4-5. Speech impairment were defined as inability to create or form speech sounds (Strand and McCauley, 2008).

### Eligibility Criteria

Inclusion: Children aged 4 to 15 years diagnosed with CP (by a pediatric neurologist who would using standardized diagnostic criteria) of any type or severity. CVI and PVD were not criteria for inclusion before randomization into the study (rational in introduction).

Exclusion: Children beyond the age criteria, with other causes of motor disorders, children whose carers refuse to participate and children with CP who have debilitating illness and require immediate medical care.

## Interventions

### Intervention Arm

The intervention was the application of carer selected tailored IQI VSS (at maximum of 8) based on the “sometimes”, “often” or “always” response to the 52 IQI questions which they consider to be the most important, relevant and practical to implement. So if any one of the 52 questions in Insight was responded to as being a problem always, often or sometimes, a group of strategies to help adapt to this particular problem was suggested and explained to the carer. If 6 questions were responded to as problematic, 6 groups of strategies would be administered to the carer. If more than 8 were responded to, the most relevant 8 problems/strategy groups chosen by the parents were selected for the carer to concentrate on. The strategies were explained at baseline and reinforced with phone calls.

### The Standard/Control Treatment Arm

The types of challenges highlighted by the IQI are not assessed or treated routinely in management of CP in our environment, in the control arm, no vision support strategies were given after the IQI had been administered. After the 6 week follow up assessment had been completed, children in the control arm were offered IQI vision support strategies based on their response to the IQI.

### Outcome

The primary outcome was change in quality of life, between baseline and follow up, assessed using the PedsQL 4.0 Generic and PedsQL 3.0 CP modules. These outcomes were all compared between the intervention and standard treatment arms at six weeks.

The secondary outcome measure for visual function was the IQI mean scores change/difference from baseline to follow up.

### Data Collection Methods

Data forms for socio-demography of carers and subjects were filled and analyzed. The PedsQL 4.0 Generic, PedsQL 3.0 CP, IQI and IQI VSS, and follow up forms were used to collect data. Data were collected at baseline and at the end of follow up after 6 weeks. Data was entered into one Microsoft excel database by a masked medical records officer who was not part of data collection.

### Quality of Life– Methodology

The quality of life methodology is described according to the format of the designers. For the PedsQL 4.0 Generic and the PedsQL 3.0 CP modules, the parent’s proxy form was used. To create Scale Scores, the mean was computed as the sum score of the items over the number of items answered (this accounts for missing data). If more than 50% of the items in the scale are missing, the Scale Score should not be computed. Imputing the mean of the completed items in a scale when 50% or more are completed was the method used.

The PedQL 4.0 Generic module has 4 subscales which identifies problems with: (1) Physical functioning, (2) Emotional functioning, (3) Social functioning and (4) School functioning.

The PedQL 3.0 CP module has 7 subscales which identifies problems in activities in everyday living, they are:(1) daily activities (2) School activities, (3) Movement and Balance, (4) Pain and Hurt, (5) Fatigue, (6) Eating activity, (7) Speech and Communication.

The school scores were not used as over 50% of children were not in school. To create the Total Scale Score, the mean is computed as the sum score of all the items over the number of items answered on all the Scales.

The higher the score report the better the quality of life.

### Sample Size

Unpublished data from a pilot study in Bangladesh was used in the sample size calculation. In this study of 180 children with CP, the visual support strategies showed an impact on quality of life, measured by PedsQL 4.0 Generic of approximately 0.3 standard deviation (SD) (Mitry et al., 2016). Using the Altman nomogram, a sample size of approximately 370 children with CP, with 185 children in each arm, is needed to detect an effect size of 0.3SD with 80% power and at a 95% confidence, allowing for 5% loss to follow up.

### Randomization: Sequence Generation

The database of children recruited with information on their unique ID, age, sex and GMFCS was sent to the data analyst in the University of Calabar Teaching Community Medicine Department at the end of the examination per local government area. The randomization sequence was generated by the data analyst (SA) using Stata 11 programming syntax for block randomization of patients into the treatment and control group. Children were stratified and blocked by age groups 4-9 and 10-15 and by Gross Motor Function Classification Score (GMFCS) Levels 1-3 and 4-5.

### Randomization: Allocation Concealment

Carers of children where contacted by an independent research clerk who was masked to baseline examination. Carers were invited to visit the same primary health center where the baseline examination was conducted. At this visit, carers assigned to the treatment arm of the trial received the intervention which was explained to them by the social workers while those in that standard arm received counseling on observation of the child’s symptoms and a final follow up call.

### Implementation/Fidelity

Carers were to identify a maximum of 8 best strategies for implementation and were to conduct the strategies three times daily. Carers were encouraged to start with the selection of 1-3 of the most important strategies to them, to start with and practice the intervention three times a day, and those which could be implemented thereafter were identified and explained with each follow up phone call. Carers were given a list of all the strategies they had selected to study further. To improve adherence and as part of the intervention, carers were contacted by phone call weekly for 6 weeks, to ask about the application of the strategy, the frequency of the application and if there were any side effects. Carers of children in intervention and control arms were unlikely to meet after the baseline assessment and allocation to arms, as each family was discharged individually and no physical group or internet based group was formed.

### Blinding/Masking

A different set of social workers conducted the post intervention interviews. Allocation was concealed to the PI and all the members of the examination team who were different from those in the intervention and follow up team.

### Follow-Up

The trial period was 6 weeks, during which phone calls were made to monitor the progress of implementation of the visual support strategies, to identify additional new strategies for implementation for the following weeks, and to remind parents to visit the primary health center for the final follow up. Follow-up interim calls were performed weekly for 6 weeks in the intervention arm. The standard treatment arm received a single call at the 6th week as a reminder for the final follow up visit. Final interviews and data collection were conducted in the primary health centers for those that presented and in homes for those that could not present.

### Informed Consent

The study was performed in accordance with the Helsinki declaration and approved by London School of Hygiene and Tropical Medicine. A written information sheet was read out and informed consent was obtained from all subjects under 18, from a parent and/or legal guardian.

### Statistical Methods

Statistical analyses were conducted with STATA (version 15.1) Analysis of data for parametric or non-parametric distribution was initially done. The *T*-tests was used to detect difference in parametric data with a significance threshold at *p* = 0.05. These were used to compare intervention versus control group and also to assess the relationship between baseline characteristics and the primary outcome measure.

We analyzed by intention to treat (the treatment group was considered as all those randomized to get IQI VSS), per IQI protocol (treatment group considered as those that actually required and received at least 1 VSS) and per VSS treatment (those that actually implemented at least 1 tailored VSS based on parent report) approach (as reported in the phone calls/at follow up), for all primary outcome analysis. Descriptive statistics included means and SDs, and medians and interquartile ranges as appropriate. The number and proportions for categorical variables describing the sociodemographic details of the population by randomization were derived.

The impact of our intervention was measured using the difference in pre and post intervention scores.

The analysis of the IQI that showed responses with >/ = 50% as non-applicable and questions 9,5,15,32,33,34,45,46 were expunged.

### Ethical Approval

The study was performed in accordance with the Helsinki declaration and approved by the ethics committees of Cross River State and the London School of Hygiene & Tropical Medicine. A written information sheet was read out. Informed consent was obtained from parents and/legal guardian. Children were referred for health care services as needed.

## Results

### Participants Flow

A total of 1024 children were identified by key informants, 293(28.6%) were not brought for examination. 731 children were assessed for cerebral palsy diagnosis eligibility, 343(46.9%) did not meet the inclusion criteria for CP and 388(37.9%) were confirmed to have CP; all enrolled into the RCT, no parent declined to participate. 388 children who met the criteria for CP were then randomized; 191(49%) were allocated to intervention while 197(51%) were allocated to standard intervention. Of the 191 that were allocated to intervention, 44(23%) of children did not meet the criteria for requiring VSS treatment as they did not answer, ‘always’, ‘often,’ or ‘sometimes’ to any of the Insight Questions Inventory questions.

Follow up phone calls in the intervention arm was made in the following frequency: week 1: 99(26%), week 2: 96(25%), week 3: 82(21%), week 4: 71(18.3%), week 5: 55(14%) and week 6: 37(10%). However, the key informant method ensured that the key informants knew each child’s home and was able to ensure they attended the follow up.

383 (98%) children completed the study protocol, and analyses performed for the primary and secondary outcomes.

### Recruitment

Children were recruited per local government area into the study from December 2016, and allocated to an intervention arm or a standard treatment arm (Table 1). The study data collection was closed in December 2018.

**TABLE 1:** Sociodemographic and clinical characteristics of the intervention and no intervention arms (*N* = 388).

Characteristics		Intervention arm		Control arm	
		No	%	No	%
**Total**		**191**	**100.00**	**197**	**100**

Local government area					
	Abi	3	1.6	2	1.0
	Akamkpa	6	3.1	8	4.1
	Akpabuyo	8	4.2	13	6.6
	Bakassi	6	3.1	5	2.5
	Bekwara	15	7.8	13	6.6
	Biase	8	4.2	11	5.6
	Boki	15	7.8	16	8.1
	Calabar Municipality	7	3.7	3	1.5
	Calabar South	13	6.5	19	9.6
	Ikom	10	5.2	10	5.2
	Etung	7	3.7	8	4.1
	Obanliku	13	6.8	14	7.0
	Obubra	9	4.7	11	5.6
	Obudu	15	7.8	12	6.0
	Odukpani	3	1.8	4	2.0
	Ogoja	25	13.5	19	9.6
	Ugep	8	4.2	8	4.1
	Yala	20	10.5	21	10.6
Residence					
	Urban	21	11.0	23	11.7
	Rural	170	89.0	174	88.3
Sex					
	Male	120	62.8	109	55.3
	Female	71	37.2	88	44.7
Child’s age					
	Mean (SD)	9.08	4.0	9.2	4.0
	Median (IQR)				
	<9 years	98	51.3	114	57.9
	9 + years	93	48.7	83	42.1
GMFCS					
	1	35	18.3	35	17.8
	2	80	41.9	76	38.6
	3	22	11.5	32	16.2
	4	30	15.7	24	12.2
	5	24	12.6	30	15.2
GMFCS					
	Ambulatory	137	71.7	143	72.6
	Non-ambulatory	54	28.3	54	27.4
Anatomic					
	Monoplegia	18	9.4	13	6.6
	Triplegia	26	13.6	21	10.7
	Diplegia	10	5.2	9	4.6
	Hemiplegia	70	36.6	80	40.6
	Tetraplegia	67	35.1	74	37.6
CP Type					
	Spastic	137	71.7	134	68.0
	Ataxic	20	10.5	18	9.1
	Dystonic	10	5.2	8	4.1
	Unclassified	13	6.8	19	9.6
	Choreoathetoid	11	5.8	18	9.1
Visual acuity (Mirror Test)					
	Normal	92	48.2	83	42.1
	Visual impairment	99	51.8	114	57.8
PVD(IQI)					
	PVD	147	77	188	95.4
	No PVD	44	23	9	4.6
PVD(tests)					
	PVD	86	48.6	91	51.4
	No PVD	105	51.4		48.6

### Baseline Data

Baseline demographic and clinical characteristics of each group can be seen in Table 1.

Table 2 shows the distribution of the place of residence and father and mothers’ educational status of children with CP. 89% of children were from rural areas, 30% of mothers and 29% of fathers were subsistent farmers.

**TABLE 2:** Distribution of the place of residence and father and mothers’ educational status of children with cerebral palsy (*N* = 388).

Variables	No	Percent
Fathers age (*n* = 353)	Mean age 42.7(SD 15.5); Median 40;(IQR35,50)	
Mothers age (*n* = 373)	Mean age 34.3(SD 8.4); Median 32 (IQR 29, 40)	
Place of residence		
Urban	44	11.3
Rural	344	88.7
Educational status		
Fathers educational status		
No formal education	19	4.9
Incomplete primary education	7	1.8
Completed primary	56	14.4
Completed junior secondary	15	3.9
Completed senior secondary	129	33.2
Post-secondary education	79	20.4
Post graduate education	2	0.5
Could not be ascertained	81	20.9
Total	388	100
Mothers educational status		
No formal education	17	4.4
Incomplete primary education	16	4.1
Completed primary	81	20.9
Completed junior secondary	26	6.7
Completed senior secondary	150	38.7
Post-secondary education	60	15.5
Post graduate education	1	0.3
Could not be ascertained	37	9.5
Total	388	100
Occupational/Skills		
Mother		
Unskilled (subsistence farmers)	118	30.4
Semiskilled	233	60.0
Civil/Public servant	28	7.2
Professional	9	2.3
Father		
Unskilled (subsistence farmers)	112	28.9
Semiskilled	196	50.5
Civil/Public servant	66	17.0
Professional	14	3.6
Income/month		
Fathers income *n* = 353	Mean $13.4; Median 0; IQR(0, 0)	
Mothers income *n* = 373	Mean $13.1; Median 0; IQR(0, 5.3)	

A total of 331(85.3%) children had speech impairment and 173(44.6%) had communication impairment.

A total of 335/388(86.3%) children had at least one symptom of PVD in this population, 147/191(77.0%) in the intervention arm which required the administration of at least one Insight Questions visual support strategy and 188/197(95%) in the control arm, *p* = 0.0012.

We assessed 6 factors at baseline for their effect on QoL; residence (urban vs. rural), sex (male vs. female), age (< 9 years vs. > 9 years), type of CP (spastic vs. others), GMFCS (ambulatory I-III- vs. non-ambulatory IV-V), and presence or absence of PVD (according to the IQI). Baseline QoL comparisons showed that those with non-spastic CP had a better QoL (mean 43.1 vs. 39.2, *p* = 0.0337 for PedsQL 4.0 Generic and mean 58.2 vs. 48.8, *p* = 0.001 for 3.0 PedsQL CP); also for PedsQL 3.0 CP only, children older than 9 years had a higher mean and better QoL (56.9 vs. 45.4 *p* < 0.001).

There was a baseline difference in QoL between the two arms with the PedsQL 4.0 Generic, with children in the treatment arm having better QoL (42.1 vs. 38.7, *p* = 0.0411).

### Numbers Analyzed

Three hundred and eighty-eight children were randomized and 191/388 (49%) allocated to intervention and 197/388(51%) allocated to standard treatment representing the number in the intention to treat analysis.

For the per IQI protocol analysis, 147/191(77%) met the criteria for the IQI protocol administration of intervention, as forty-four children in the intervention group did not have symptoms of PVD in any subdomain and therefore did not get strategies. For the per VSS treatment analysis, there were 91/191(48%) participants randomized to treatment who actually implemented the strategies three times a day.

The mean number of strategies assigned to each family was 3.7(SD 2.9).

The first three choices of strategies with the highest frequencies chosen by parents were to Insight Questions and strategy numbers 17:100/147(68%); If the child has difficulty catching a ball:

➊ Practice catching skills with your child by throwing a balloon to each other. The balloon will move slower than a ball and may be easier for your child to catch.➋ Put a little bit of rice/water in the balloon. The balloon will make a noise as it moves so your child can hear where it is. ➌ Use large, brightly colored balls when playing catch or other ball games with your child.➍ Use balls with sound or light effects when playing catch or other ball games with your child.

Number 4:52/147(35%); If the child appears to ‘get stuck’ at the top of a slide or hill:

➊ Encourage your child to practice around the house trying to cross small gutters and playing on small play slides and/or by lying on his tummy on a scooter board or skate board. Some children choose to go down slides head first. Do not stop this, but make sure it is safe. Children may do this because the upper part of their field of view is being used in this situation.➋ Give additional verbal information.

Number 37:40/147(27%).

If the child reacts angrily when other restless children cause distraction:

➊ If possible, take other distractions away from your child’s work area. i.e., sound, movement.➋ Let your child use head phones or ear plugs so noise does not disturb him.➌ See how your child gets on sitting at a separate desk at the end of the group. This may give him more space without leaving him out of the group.

### Outcomes and Estimation

For the primary outcome analysis, the results of ‘intention-to-treat,’ ‘per IQI protocol’ and ‘per VSS treatment’ analysis did not show any significant difference between intervention and control groups, in change or improvement in the total quality of life in children using either the generic or the PedsQL-CP tool using the unpaired t test. (Tables 3, 4 shows the intention to treat analysis, per IQI protocol and per VSS implementation for the Total CP generic and CP scores). Testing indicated that all the data were parametric so *t*-test were used throughout.

**TABLE 3:** Primary outcome measure, total PedsQL 4.0 Generic scores using intent-to-treat (*N* = 388), per protocol (*N* = 344), per treatment analysis (*N* = 288).

Variable	Intention to treat			Per IQI protocol			Per VSS treatment		
	Intervention arm	Control arm	*P* value (*t* test)	Intervention arm	Control arm	*P* value (*t* test)	Intervention arm	Control arm	*P* value (*t* test)
Total PedsQL 4.0 baseline								0.3234	
N	191	197		147	197		91	197	
Mean (SD)	42.1(SD 19.5)	38.7(SD 18.6)		43.2(SD 18.3)	38.7(SD 18.7)		41.2(SD 17.5)	38.7(SD 18.6)	
Median (IQR)	41.7(25, 56.25)	37.5(25, 54.2)		43.7(27.1, 56.2)	37.5(54.2)		41.7(27.1, 52.1)	37.5(25, 54.2)	
Total PedsQL 4.0 follow up									
N	157	157		125	157		91	197	
Mean (SD)	45.1(SD 22.9)	44.1(SD 21.6)		45.5(SD 21.9)	44.1(SD 21.6)		41.2(SD 17.5)	38.7(SD 18.6)	
Median (IQR)	43.7(27.1, 58.3)	41.7(29.2, 58.3)		43.7(29.5, 58.3)	41.6(29.2, 58.3)		41.7(27.1, 52.1)	37.5(25, 54.2)	
Total PedsQL 4.0 difference			0.943			0.9317			0.100
N	157	157		125	157		81	157	
Mean (SD)	2.3(SD 11.8)	5.0(SD 17.8)		2.3(SD 11.8)	5.0(SD 17.8)		2.3(SD 10.0)	5.0(SD 17.8)	
Median (IQR)	9(0, 0)	0(0, 0)		0(0, 0)	0(0, 0)		0(0, 0)	0(0, 0)	

**TABLE 4:** Showing the results for the primary outcome measure on the total PedsQL 3.0 CP scores using intent-to-treat (*N* = 382), per protocol (*N* = 339) and per treatment analysis (*N* = 283).

Variable	Intention to treat			Per IQI protocol			Per VSS treatment		
	Intervention arm	Control arm	*P* value (*t* test)	Intervention arm	Control arm	*P* value (*t* test)	Intervention arm	Control arm	*P* value (*t* test)
Total PedsQL 3.0 baseline									
N	189	193		146	193		90	193	
Mean (SD)	53.5(SD 26.5)	49.8(SD 27.6)		53.9(SD26.3)	49.8(SD27.6)		52.4(SD 26.7)	49.8(SD27.6)	
Median (IQR)	55.5(34.7, 76.1)	45.2(27.4, 74.2)		53(34.7, 76.6)	45.2(27.4, 74.2)		47.6(33.9, 74.2)	45.2(27.4, 74.2)	
Total PedsQL 3.0 at follow up									
N	164	162		130	162		86	162	
Mean (SD)	60.4(SD27.2)	54.4(SD 27.5)		62.3(SD 26.9)	54.4(SD27.5)		61.6(SD 27.8)	54.4(SD27.5)	
Median (IQR)	61.7(42.2, 83)	53, 2(32.2, 77.4)		63.3(43.5, 87.1)	53.2(32.2, 77.4)		64.9(41.9, 87.1)	53.2(32.2, 77.4)	
Total PedsQL 3.0 difference			0.287			0.163			0.908
N	163	161		130	161		86	161	
Mean (SD)	6.3(SD 22.2)	4.9(SD 23.2)		7.5(SD 22.4)	4.9(SD 23.2)		9(SD 9)	4.9(SD 23.2)	
Median (IQR)	0(−2.4, 19.5)	0(0, 13.7)		0(−1.6, 20.1)	0(0, 13.7)		0(−2.4, 27.4)	0(0, 13.7)	

Table 5 shows the subdomains of speech and communication, where there was a better improvement in quality of life in the intervention group compared to the control group, analyzed by intention to treat and per IQI protocol. (*p* = 0.035 and *p* = 0.006).

**TABLE 5:** Primary outcome measure, pediatric quality of life cerebral Palsy speech and communication subdomain scores for using intent-to-treat (*N* = 310), per protocol (*N* = 247), per treatment analysis (*N* = 227).

Variable	Intention to treat			Per IQI protocol			Per VSS treatment		
	Intervention arm	Control arm	*P* value (*t* test)	Intervention arm	Control arm	*P* value (*t* test)	Intervention arm	Control arm	*P* value (*t* test)
PedsQL 3.0 Speech and communication at baseline									
N	154	156		118	156		71	156	
Mean (SD)	44.1(SD 40)	42.9(SD 41)		42.3(SD 40)	42.9(SD 41)		43.3(SD 41.4)	42.9(SD 41)	
Median (IQR)	37.5(0, 81.2)	31.2(0, 94)		34.3(0, 81.2)	31.2(0, 94)		37.5(0, 93.7)	31.2(0, 94)	
PedsQL 3.0 Speech and communication at follow-up									
N	139	136		110	136		71	136	
Mean (SD)	51(SD 43.1)	42.2(SD 41)		53.6(SD 43.6)	42.2(SD 41)		55(SD 44.4)	42.2(SD 41)	
Median (IQR)	50(0, 100)	25(0, 93.7)		50(0, 100)	25(0, 93.7)		62.5(0, 100)	25(0, 93.7)	
PedsQL 3.0 Speech and communication difference									
N	133	130	0.0350	105	130	0.006	66	130	0.994
Mean (SD)	7(SD 35.2)	−0.64(SD 32)		10.5(35.7)	−0.64(SD 32)		12.4(SD 46.8)	-0.64(SD 32)	
Median (IQR)	0(0, 18.75)	0(0, 0)		0(0, 025)	0(0, 0)		0(0, 31.2)	0(0, 0)	

The secondary outcome measure (the Insight Questions Inventory) is reported in Table 6 as intention-to-treat, per IQI protocol or per VSS implementation. There was no overall difference between intervention and control groups.

**TABLE 6:** Secondary outcome measure of visual function using the Insight Questions Inventory using intent-to-treat (*N* = 383), per protocol (*N* = 341), per treatment analysis (*N* = 285).

Variable	Per treatment			Per IQI protocol			Per VSS treatment		
	Intervention arm	Control arm	*P* value (*t* test)	Intervention arm	Control arm	*P* value (*t* test)	Intervention arm	Control arm	*P* value (*t* test)
Total IQI score at baseline									
N	188	195		146	195		90	195	
Mean (SD)	1.5 SD(0.5)	1.7 SD(0.7)		1.6 (SD 0.48)	1.7 (SD74)		1.64(SD 0.54)	1.67(SD 0.74)	
Median (IQR)	1.3(1.2, 1.7)	1.4(1.2, 1.9)		1.4 (1.2, 1.7)	1.4(1.2, 1.9)		1.5(1.3, 1.8)	1.4(1.2, 2.0)	
Total IQI follow up scores									
N	188	192		146	192		90	192	
Mean (SD)	1.2 SD(0.62)	1.3 SD(0.8)		1.25 (SD0.57)	1.3 (SD 0.8)		1.36(SD 0.51)	1.34(SD 0.81)	
Median (IQR)	1.3(1.0, 1.5)	1.2(1.0, 1.1.6)		1.2(1.0, 1.5)	1.2(1.0, 1.66)		1.3(1.0, 1.56)	1.3(1, 1, 1.67)	
Total IQI scores of difference			0.322			0.659			0.567
N	188	192		146	192		90	192	
Mean (SD)	−0.27 SD (0.61)	−0.3 SD (0.8)		−0.33(SD 0.6)	−0.30(SD0.8)		−0.29(SD 0.51)	−0.30(SD 0.79)	
Median (IQR)	−0.13(−0.4, 0)	−0.12(−0.4, 0)		−0.15(−0.3, −0.5)	0(−0.1, 0)		−0.17(−0.4 −0.05)	0(−0.17, 0)	

### Adverse Events

Two patients died in the standard treatment arm and one in the intervention arms of the study due to complications of CP. There were no side effects reported from any group.

## Discussion

In this community based RCT, visual support strategies aimed at compensating for visual perceptual problems identified by the Insight Questions inventory (IQI VSS) were not shown to significantly improve the overall quality of life of children. The IQI tool did not function effectively for this population. It did not elicit sufficient positive responses in children who seemed to have evidence of PVD using some basic objective tests (Duke et al., 2020). Since the IQI VSS depends on such positive responses this would have limited its effectiveness. IQI did seem to be effective, in a similar population in Bangladesh, in eliciting symptoms which did relate to quality of life; and IQI VSS have shown some promise in improving quality of life in a UK hospital based study of children with CVI (Tsirka et al., 2020). Although it has been successful in other studies it should be noted that the IQI questions taken individually are not specific for CVI or PVD: they could be answered positively by children with ocular VI or pure motor impairment.

Further work is required refining this tool for this population including investigation of which questions are appropriate, for children with CP/comorbidities, for families in more rural environments, and for parents with a range of education levels. It is possible that a questionnaire approach is not the best one for this population. Separate from how the IQI performed we did find evidence of under recognition/reporting of visual morbidity by the carers of these children (Duke et al., 2020). In retrospect it could be argued that we should have screened for CVI or PVD before recruiting e.g., perhaps had a cut off requiring a certain number of positive IQI responses to be eligible for recruitment. Our assumption was that almost all children with CP have some level of PVD but this proved not to be the case; almost a quarter did not receive any strategies which resulted in an underpowered trial. The results might have been influenced by chance differences between the 2 groups at baseline. There was a higher proportion of PVD in the control group compared to the intervention group. Also the treatment group had better scores PedsQL 4.0 scores at baseline though this was a small difference and would lose significance after adjustment for multiple comparison.

Another reason for the lack of effect could have been poor adherence to the strategies. The rate of successful follow-up calls dropped substantially across the 6-weeks intervention period, it is possible that parent implementation of strategies decreased and that adherence might have been over reported.

Improvement due to the use of IQI visual support strategies, in the health related quality of life in the CP speech and communication domain was suggested across the intention to treat and per IQI protocol. However, this may be a chance finding as our analysis was not adjusted for multiple comparisons. (Since there were 2 PedsQL modules each with subsections, a threshold value *p* value of 0.05, for instance, would drop to 0.003) The impact of the VSS on speech and communication needs further investigation before drawing strong conclusions and should be further investigated since a large proportion of children in this population had speech impairment (85%) and 45% had communication difficulties. It is well reported that these are common problems in children affected by CP (Pennington et al., 2004). Various strategies have been tried, but evidence of their effectiveness is limited (Pennington et al., 2004).

A surprising finding was that quality of life seemed to improve more in the control arm than the intervention arm when the PedsQL 4.0 Generic score was used whereas the reverse was true when the PedsQL CP 3.0 module was used. The differences were not significant and may be due to chance but this difference in trend is notable and may be because of the differences in the 2 tools with the CP module being more physical disease focused and easier for parents to relate with their child’s problems. Another possible reason for the discrepancy between the 2 PedsQL versions is that the main benefit was seen in the speech and communication domain of the PedsQL 3.0 CP tool which is not present in the generic version. Some children appeared to have a decrease in quality of life in the 4.0 Generic module following the intervention. It is possible that having undergone lengthy assessments and not received any medical treatment or glasses immediately, carers may have felt a lack of intervention or improvement and reflected this in their answers. In addition, they may have thought more (after the first experience of answering the PedsQL questionnaires) about problems children have, so give more negative results second time around, a possible situation of investigation fatigue (this may have also contributed to the lack of improvement in IQI scores in the treatment group, where previous work did find such an improvement) (Tsirka et al., 2020).

### Limitations of the Study

A rigorous RCT was conducted in a field where there is little RCT evidence on which to base management of this condition. However, the assessment and intervention tool did not seem to work well in this population, despite having worked well in a range of geographic populations previously. IQI scores and the number of children receiving IQI VSS were smaller than predicted, likely underpowering the trial. More cultural adaptation and piloting would have been beneficial such as checking whether the questions were appropriate (for children with CP/comorbidities, for families in more rural environments and for parents with the education levels noted).

## Conclusion

The use of the IQI and IQI VSS for the treatment of PVD in children with CP in this population did not show any superiority over current standard measures of treatment. The study suggests that further investigation and refinement of this type of intervention is required for this population. There was a suggestion of a positive effect in the area of speech and communication related quality of life.

## Data Availability Statement

The raw data supporting the conclusions of this article will be made available by the authors, without undue reservation.

## Ethics Statement

The studies involving human participants were reviewed and approved by London School of Hygiene and Tropical Medicine Ethics Committee and the Cross River State Ethics Committee. Written informed consent to participate in this study was provided by the participants’ legal guardian/next of kin.

## Author Contributions

RD and RB contributed to conception, design, execution, supervision, analysis and writing of the manuscript. TC and SA contributed to design and execution. MK and KB contributed to design, supervision, analysis and writing. All authors contributed to the article and approved the submitted version.

## Conflict of Interest

The authors declare that the research was conducted in the absence of any commercial or financial relationships that could be construed as a potential conflict of interest.

## Publisher’s Note

All claims expressed in this article are solely those of the authors and do not necessarily represent those of their affiliated organizations, or those of the publisher, the editors and the reviewers. Any product that may be evaluated in this article, or claim that may be made by its manufacturer, is not guaranteed or endorsed by the publisher.
