# Supplementary material for: The Effect of Insight Questions Inventory and Visual Support Strategies on Carer-Reported Quality of Life for Children With Cerebral Palsy and Perceptual Visual Dysfunction in Nigeria: A Randomized Controlled Trial
Source: Front Hum Neurosci. 2021 Nov 16;15:706550. doi: 10.3389/fnhum.2021.706550 (PMC8636698; doi:10.3389/fnhum.2021.706550)
Supplement: Supplementary file 1 [file Data_Sheet_1.pdf]

Supplementary material 1: Modified Insight Questions Inventory and visual support strategies (Nigerian version)

About your child

Child's forename:	Surname:	
D.O.B.:	Gender:	
Instructions to parents		
These questions are designed for a range of ages, so some questions may seem odd. Your child may have difficulty with some behaviours listed below but not others – this is normal.		
For each of the items listed, please could you tick on the box next to it and select which best fits with your child's present behaviour: never/ rarely/ sometimes/ often/ always/ not applicable (NA).		

1 Does your child fall or stumble onto things on the floor?

2 Does your
child find it
hard to walk
down the hill
or stairs?

3 Does your child want to
fall or does it seem as if
your child does not notice
when the foot path or
pavements go up or down?

4 Does your child get frightened
not knowing what to do at the
top of a slide or hill or in front
of a gutter?

5 Does your child look down
when they cross floor
boundaries, for example where
tiles meets the cement floor?

6 Does your child leave food on their plate? If so, is this on the near or far side?

7 Does your child leave food on their plate? If so, is this on the left or right side?

8 Does your child have any problem stepping into the bath, apart from any problems with being able to stand by himself well-balance?

9 Does your child find it hard/difficult to find the beginning of a line or the next word when reading, or miss pictures or words on one side of a page (left /right)?

10 Does your child walk, move out in front of traffic (coming from left/ right / both)?

11 Does your child hit his/her body into door frames or partly open doors (left/right/both) when trying to pass through?

12 Does your child have difficulty/hard seeing things from a moving vehicle?

13 Does your child find it hard to identify things (or know what has just passed by) that are moving pass quickly, such as children, small animals?

14 Does your child find it hard to see moving water in a container i.e. to know when the cup is full?

15 Does your child find it hard to follow the arrow head on the computer screen?

16 Does your child avoid watching fast moving video CD, and prefer to watch slow moving video CD programmes?

17 Does your child find it hard to catch a ball?

18 When walking, does your child hold onto your clothes, dragging down?

19 Does your child find uneven ground hard to walk over?

20 Does your child run into low furniture, for example a small stool?

21 Does your child get angry if furniture is moved?

22 Does your child explore floor boundaries (for example, where

tiles meets a cement floor) with their foot before crossing

23 Does your child reach incorrectly for objects (i.e. reaches beyond or around the object or when picking it up, grasp incorrectly, missing or knocking the object over)?

24 Does your child find it hard to see something pointed out in the distance?

25 Does your child find it hard to know a relative or friend who is standing in a group?

26 Does your child find it confusing or difficult when playing team games with a lot of players, especially if there are a lot of players and everyone is moving?

27 Does your child have difficulty finding an item in the kitchen or cupboard shelf e.g. finding the salt or pepper?

28 Does your child find it hard to find an item of clothing in a pile of clothes?

29 Does your child find it hard to find something they want in a full toy cartoon, shoe in a box of shoes etc?

30 Does your child find it hard to find anything when the object is in a place with the same color of the place? E.g. a white tee shirt on a white bedsheet?

31 Does your child get lost in places where there is a lot to see, e.g. in a crowded market or church?

32 Does your child find copying words or drawings time consuming and difficult?

33 Does your child have difficulty reading crowded text on paper or on a computer screen, but can cope better if some of the text is covered or taken away?

34 Does your child find it hard to notice or look for letters on a keyboard but knows the alphabet?

35 Does your child sit closer to the television than about 30 cm?

36 Does your child find it difficult
to concentrate or focus or
perform a task for more than 5
minutes, or after being distracted
do they find it difficult to get
back to what they were doing?

37 Does your
child react
angrily when
other children
cause
distractions or
making noise
or movements
all around?

38 Does your child hit into things
when they are walking and
talking at the same time?

39 Does your child miss
objects that are obvious to
you because they are
different from their

background and seem to
'jump out' e.g. a bright ball
in the grass?

40 Does your
child get angry
in busy places,
such as
markets or
church?

41 Does your
child find it
difficult to
recognise close
relatives in real
life?

42 Does your
child find it
hard to know
who their
aunties and
uncles' or
sisters and

brothers are from photographs?

43 Does your child mistakenly identify strangers as people known to them?

44 Does your child have difficulty understanding the meaning on your face?

45 Does your child find it hard to name common colours?

46 Does your child have difficulty naming basic shapes(call the

names of the shapes) such as squares, triangles and circles?

47 Does your child find it hard to recognise well known objects such as the family house or the church building?

48 Does your child find it hard to find their way around a well known environment, for example their home or church?

49 Does your child find it hard to recognise an object if it is partially hidden or viewed from an unusual angle (such as a shoe under the bed with only the toe showing)?

50 Does your child find it hard to identify which shoe is right and which shoe is left?

51 Does your child find it hard to recognise people, or words, or objects if they are changed in any way from what they are used to, for example font of text, hairstyle?

52 Does your child find it difficult to identify where sound is coming from, for example if they shout for you in the house do you have to clearly tell them which room you are in, as they are unable to work out where you are from your voice alone?
